# Real-time characterization of the molecular epidemiology of an influenza pandemic

**DOI:** 10.1098/rsbl.2013.0331

**Published:** 2013-10-23

**Authors:** J. Hedge, S. J. Lycett, A. Rambaut

**Affiliations:** 1 Institute of Evolutionary Biology, University of Edinburgh, Ashworth Laboratories, Edinburgh, UK; 2 Fogarty International Center, National Institutes of Health, Bethesda, MD, USA

**Keywords:** Bayesian phylogenetics, influenza, pandemic, parameter estimation, real-time

## Abstract

Early characterization of the epidemiology and evolution of a pandemic is essential for determining the most appropriate interventions. During the 2009 H1N1 influenza A pandemic, public databases facilitated widespread sharing of genetic sequence data from the outset. We use Bayesian phylogenetics to simulate real-time estimates of the evolutionary rate, date of emergence and intrinsic growth rate (*r*_0_) of the pandemic from whole-genome sequences. We investigate the effects of temporal range of sampling and dataset size on the precision and accuracy of parameter estimation. Parameters can be accurately estimated as early as two months after the first reported case, from 100 genomes and the choice of growth model is important for accurate estimation of *r*_0_. This demonstrates the utility of simple coalescent models to rapidly inform intervention strategies during a pandemic.

## 1. Introduction

When the swine-origin influenza A virus (A(H1N1)pdm09) was detected in April 2009, rapid characterization of its transmission potential and pathogenicity was urgently required for determination of appropriate interventions [1]. Early estimates of its emergence and transmission using phylogenetic analysis of genetic sequence data were reported within just three months of detection [2,3]. Such analyses are possible owing to rapid accumulation of genetic variation within the virus population, enabling its evolution to be modelled on an epidemiological timescale [4].

Here, we determine the efficiency with which Bayesian phylogenetics based on coalescent processes can estimate the evolutionary rate, date of emergence and intrinsic growth rate, *r*_0_, of A(H1N1)pdm09 using whole-genomes. The evolutionary rate provides an indication of the adaptive potential of a virus in a new host population. Accurate estimation is required for inferring divergence times and population size changes. The time of the most recent common ancestor (TMRCA) of a random sample of viruses provides an upper bound to the date of emergence of an epidemic. We use simple parametric growth models to estimate *r*_0_ as a measure of the relative ease with which A(H1N1)pdm09 spread through a host population.

## 2. Material and methods

We downloaded all available A(H1N1)pdm09 whole-genome sequences sampled April–December 2009 from the EpiFlu database hosted by the Global Initiative on Sharing All Influenza Data (GISAID; platform.gisaid.org) on 26 April 2010 (see the electronic supplementary material, table S1). We analysed whole-genomes (by concatenating segments) to maximize genetic variation in the dataset and only included North American samples to limit spatial heterogeneity in viral population structure. We removed all isolates sampled from a non-human host, missing an exact sampling date, or with sequencing coverage less than 80% for any genome segment. To minimize the effect of epidemiologically linked cases, which may confound assumptions of the coalescent, we subsampled one isolate/location/day [5,6], resulting in a dataset of 328 sequences. After aligning, we trimmed sequences to 13 158 bp.

We carried out Bayesian phylogenetic analysis of the entire dataset in BEAST v. 1.7.4 [7–9], using the GTR+*Γ* nucleotide substitution model and uncorrelated lognormal relaxed molecular clock, which had greater Bayes factor support than a strict clock (BF = 3.65) [10,11]. The clock used a gamma-distributed prior on the mean evolutionary rate, with a mean of 1 substitution/site/year (*k* = 0.001, *θ* = 1000) and exponentially distributed prior on the standard deviation (*μ* = 0.333). To model the demographic history of the virus population, we used the non-parametric Gaussian Markov random fields Bayesian skyride model [12], which specifies the prior on the TMRCA. We performed four independent Markov chain Monte Carlo runs of 100 million steps to achieve good mixing, sampling trees every 10 000 steps and combining runs after removing 10% burn-in.

To investigate how accurately and precisely Bayesian phylogenetics can estimate the evolutionary rate, date of emergence and *r*_0_ throughout the pandemic, we extracted nine subsets of sequences, each with an increasingly longer temporal range and size. This sampling strategy is akin to carrying out phylogenetic analyses using all genome data available at the end of each month between April and December 2009. Given the increasing capacity with which sequencing can be performed, we included all data available from GISAID on 26 April 2010 to estimate parameters from the maximum amount of sequence data that could have potentially been available if samples were sequenced immediately. We used the same evolutionary models as above but replaced the skyride model with either an exponential or logistic growth model [13]. Here, we use the TMRCA to represent the date of emergence of the virus into the larger human population, assuming a single initial case.

We quantified the relative fit of both growth models by comparing their marginal likelihoods as Bayes factors. The marginal-likelihood measures the average fit of a model to the data and we estimated this using a recently described path sampling procedure [11].

## 3. Results

**Figure 1.**
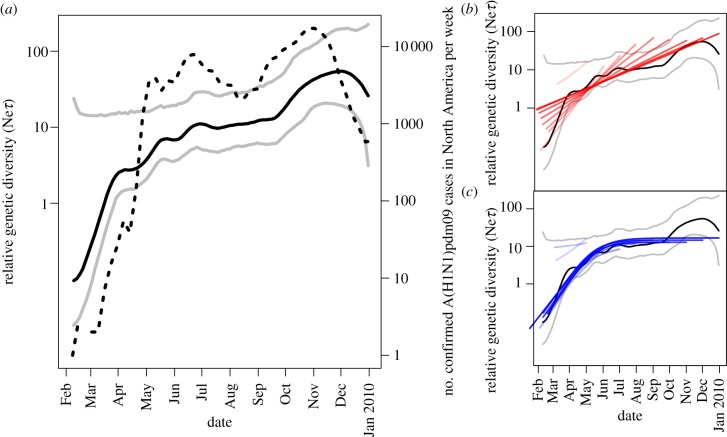
Bayesian skyride reconstruction of the demographic history of A(H1N1)pdm09 in North America until December 2009. Mean genetic diversity (solid black) with corresponding 95% BCI (grey) are shown in (*a*–*c*). Incidence rate (number of new A(H1N1)pdm09 cases confirmed by the WHO/week; dashed) is plotted on secondary axes in (*a*). Similar reconstructions from analysis of the nine cumulative datasets under the (*b*) exponential and (*c*) logistic growth models are plotted with saturation increasing with dataset size in each analysis.

The skyride plot in figure 1 shows that the reconstructed past population dynamics of A(H1N1)pdm09 closely follows the number of newly confirmed A(H1N1)pdm09 cases per week (accessed via FluNet; http://who.int/influenza/gisrs_laboratory/flunet/), used here as a measure of incidence rate. This plot captures the exponential growth phase of the first pandemic wave, the plateau in genetic diversity and the growth phase during the second pandemic wave.

**Figure 2.**
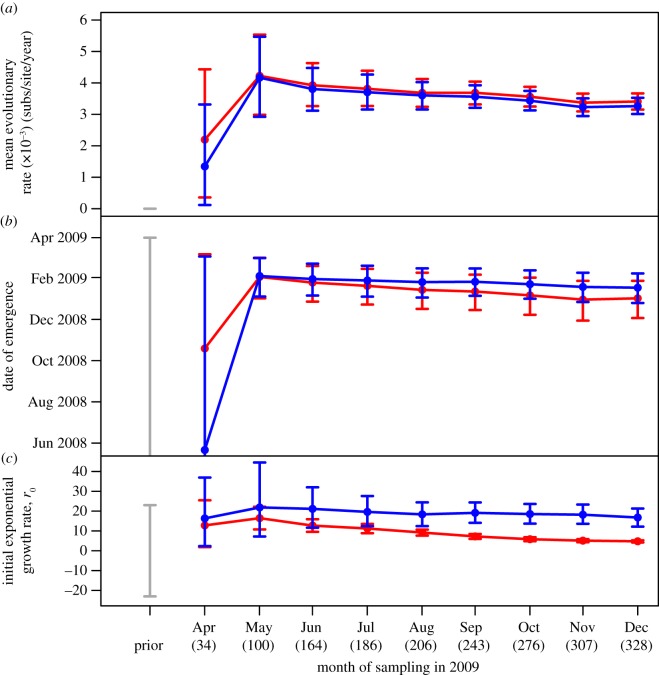
(*a*) Mean evolutionary rate, (*b*) date of emergence and (*c*) *r*_0_ estimates from Bayesian phylogenetic analysis of A(H1N1)pdm09 whole-genomes sampled cumulatively at the end of every month between April and December 2009 across North America. Exponential (red) and logistic (blue) growth models were used in analyses of each dataset. Error bars represent 95% BCI. Dataset size is displayed underneath month names in brackets.

The evolutionary rate and date of emergence estimated from the first 34 sampled genomes have wide 95% Bayesian credible intervals (BCI) under both growth models, representing high uncertainty associated with the small sample size (figure 2). Precision increases when the dataset size is increased threefold with the addition of sequences sampled during May, from which the date of emergence is estimated to be 2 February 2009 (95% BCI: 12 January 2009, 2 March 2009) and evolutionary rate 3.93 × 10*^−^*^3^ substitutions/site/year (95% BCI: 2.99, 5.53 × 10*^−^*^3^) with a standard deviation of 0.24 (95% BCI: 8.9 × 10*^−^*^6^, 4.5 × 10^−1^) under the exponential growth model. The date of emergence remains roughly consistent at later time-points and any further increase in precision is limited by the lack of alternative independent loci. Conversely, the mean evolutionary rate estimates tend to decrease with the addition of data over time, suggesting that many early deleterious/neutral mutations may have later been purged from the population through purifying selection [3,5,14].

In contrast to either of the other parameters, the choice of growth model has a considerable effect on *r*_0_ estimation (figure 2). By the end of April, both growth models fail to estimate *r*_0_ with sufficient precision to discriminate between slow and rapid epidemic growth because of the small number of sequences sampled. However, uncertainty rapidly reduces by approximately 50% under the exponential growth model with the addition of 66 sequences in May. In comparison, precision remains low during the first three months under the logistic growth model, representing over-parametrization of the model with smaller datasets. The exponential growth model consistently estimates *r*_0_ with greater precision than the logistic model early in the pandemic, once data sampled after May are included in the analysis. Genetic diversity plateaus around June and the exponential growth model inappropriately adjusts for this by lowering the *r*_0_ estimate (figure 1*b*). As the logistic growth model accommodates for this plateau, the accuracy of *r*_0_ estimates remains largely unaffected by the inclusion of data from the second wave (figure 1*c*). A Bayes factor test favours the exponential over the logistic model until June, when support switches and increases as data sampled throughout the following months are included in the analysis (table 1).

**Table 1.** Log-marginal likelihoods of both growth models used to analyse the nine subsets of sequences with increasing temporal ranges. The preferred model for each dataset (values in italics) was determined using a Bayes factor test, in which the exponential growth model was the null model.

last month sampled in dataset	no. sequences in dataset	log-marginal likelihood		Bayes factor
		exponential growth model	logistic growth model	
April	34	−*19697*.*73*	−19698.77	−1.03
May	100	−*22630.98*	−22633.16	−2.18
June	164	−26029.90	−*26029.20*	0.70
July	186	−27662.79	−*27650.54*	12.25
August	206	−29552.01	−*29543.42*	8.59
September	243	−33031.06	−*33009.23*	21.83
October	276	−36103.46	−*36078.91*	24.55
November	307	−39147.32	−*39115.72*	31.60
December	328	−41934.96	−*41912.87*	22.09

Here, *r*_0_ can be used to estimate the basic reproductive ratio (*R*_0_), which describes the average number of secondary infections arising from a primary infection [15]. For example, assuming a gamma-distributed generation time [16,17] with *μ* = 2.6 and *σ* = 1.3 (estimates from household data in the USA [18]), and *r*_0_ estimated from the first two months of data (100 sequences) under an exponential growth model, we estimate an *R*_0_ of 1.12 (95% BCI: 1.07, 1.16). This supports previous estimates from phylogenetic analyses of A(H1N1)pdm09 but is towards the lower end of estimates from incidence data sampled over similar temporal and spatial scales [2,19,20].

We investigated the effect of sample size on parameter estimation by constraining each cumulative dataset to 100 randomly selected genomes (see electronic supplementary material, figure S1). Although variation exists between the means of estimates from different random subsamples at each time-point, their 95% BCIs overlap with one another and those from analysis of the complete dataset. Additionally, the complete dataset provides only slightly higher precision of estimates of each parameter.

## 4. Discussion

Widespread genome sequencing and rapid sharing of data during the 2009 H1N1 pandemic enabled real-time characterization of an influenza pandemic for the first time [2,3,5]. Within approximately two months of the first cases (100 genomes), estimates of evolutionary rate, date of emergence and *r*_0_ from sequence data were in agreement with those from analyses of incidence data, where comparison is available [1,2,19,20]. Over a longer sampling period, parameter estimates from 100 genomes maintain similar accuracy and precision to estimates from more intensively sampled datasets. We discuss potential reasons for the general decrease in evolutionary rate observed over time, although the difference in evolutionary rates is not significant and the datasets are not independent so this result should be interpreted with caution.

The exponential growth model accurately estimates all three parameters during the exponential growth phase, although precision was low with less than 100 sequences. Once growth begins to plateau, this model should be replaced by the logistic growth model to avoid severely underestimating *r*_0_. A demographic reconstruction using a non-parametric coalescent model, such as the skyline or skyride model, can be used to reveal when exponential growth ceases [12,21]. However, these models are unable to estimate the change in relative genetic diversity between the most recent coalescent event and the youngest sample. If this time is large, the demographic plot may appear to flatten prematurely [6]. The exponential growth model is unaffected by an absence of recent coalescence events, estimating *r*_0_ from the density of early coalescent events.

Simple parametric coalescent models are powerful tools for early characterization of an epidemic, even while growth remains exponential. More complex phylogenetic models have been developed to estimate epidemiological parameters that cannot be achieved with parametric coalescent models alone [22–25]. However, the over-parametrization of early A(H1N1)pdm09 data under the logistic growth model highlights the disadvantages of using highly parametrized models during the initial stages of an epidemic. With the increasing capacity of sequencing technologies, the lag between sampling and sequencing viral genomes is expected to decrease, making earlier parameter estimation feasible in future epidemics and before alternative types of data become available.

## Data accessibility

All models, priors and settings used in this analysis are provided in the XML used as input for BEAST 1.7.4, which are available from the Dryad Digital Repository: doi:10.5061/dryad.jm858. EpiFlu accession numbers of sequences are provided in the acknowledgement of electronic supplementary material, table S1.
